# Romanian *Viscum album* L.—Untargeted Low-Molecular Metabolomic Approach to Engineered Viscum–AuNPs Carrier Assembly

**DOI:** 10.3390/plants11141820

**Published:** 2022-07-11

**Authors:** Adina-Elena Segneanu, Catalin Nicolae Marin, Dumitru Daniel Herea, Ionut Stanusoiu, Cornelia Muntean, Ioan Grozescu

**Affiliations:** 1 Institute for Advanced Environmental Research-West, University of Timisoara (ICAM-WUT), Oituz nr. 4, 300086 Timisoara, Romania; adina.segneanu@e-uvt.ro; 2 Faculty of Physics, West University of Timisoara, 300223 Timisoara, Romania; 3 National Institute of Research and Development for Technical Physics, 47 Mangeron Blvd, 700050 Iasi, Romania; dherea@phys-iasi.ro; 4 CAICON Department, University Politehnica Timisoara, 300006 Timisoara, Romania; stanusoiu@gmail.com (I.S.); cornelia.muntean@yahoo.com (C.M.); ioangrozescu@gmail.com (I.G.)

**Keywords:** low-weight metabolites, viscum, mass spectra, bioactive compounds, phyto-engineered gold nanoparticles carrier assembly

## Abstract

Viscum is one of the most famous and appreciated medicinal plants in Europe and beyond. The symbiotic relationship with the host tree and various endogenous and ecological aspects are the main factors on which the viscum metabolites’ profiles depend. In addition, European traditional medicine mentions that only in two periods of the year (summer solstice and winter solstice) the therapeutic potential of the plant is at its maximum. Many studies have investigated the phytotherapeutic properties of viscum grown on different species of trees. However, studies on Romanian viscum are relatively few and refer mainly to the antioxidant and antiproliferative activity of mistletoe grown on *Acer campestre*, *Fraxinus excelsior*, *Populus nigra*, *Malus domestica*, or *Robinia pseudoacacia*. This study reports the first complete low-molecular-weight metabolite profile of Romanian wild-grown European viscum. A total of 140 metabolites were identified under mass spectra (MS) positive mode from 15 secondary metabolite categories: flavonoids, amino acids and peptides, terpenoids, phenolic acids, fatty acids, organic acids, nucleosides, alcohols and esters, amines, coumarins, alkaloids, lignans, steroids, aldehydes, and miscellaneous. In addition, the biological activity of each class of metabolite is discussed. The development of a simple and selective phyto-engineered AuNPs carrier assembly is reported and an evaluation of the nanocarrier system’s morpho-structure is performed, to capitalize on the beneficial properties of viscum and AuNPs.

## 1. Introduction

Since ancient times, viscum was the “crown jewel” of European traditional medicine. Viscum is considered the universal healing plant, to which have been attributed to many symbols and rituals throughout the world. Practically all over the world, there are many superstitions, ceremonials, and legends associated with the mistletoe that grows on a specific host tree [1,2,3,4,5]. For instance, in Europe, a viscum-grown oak tree is said to have mystical properties (banishing witches and evil spirits, battle protection, finding treasures, love, fecundity, and so on) [1,2,3,4,5].

For the Druids, viscum was a divine tree, which grows without roots. It was evergreen, which is why it represents a connection between heaven and earth; the source of life for the tree’s spirit. 

In Japan, Ainu attributed properties similar to the viscum-grown willow tree [1,2,3,4,5]. There were rituals related to the manner and period of the viscum harvest all over Europe. 

The Druids considered that summer solstice was the moment when the maximum potential of the plant was reached. However, in many northern European countries, viscum was harvested during the winter and was associated with love, peace, protection, and fertility. 

Later, in Christian Europe, the tradition of viscum was perpetuated. Nowadays, between Christmas and New Year, the houses are decorated with viscum twigs, as a symbol of eternal life, protection, and fortune [1,2,3].

Viscum’s healing properties have been well known since ancient times. The famous scholars Hippocrates, Pliny the Elder, and then Paracelsus and Hildegard of Bingen used the plant for various diseases of the spine, liver, infertility, epilepsy, and ulcers [6].

Afterwards, viscum was considered a remedy for pain, parotitis, epilepsy, edema, cardiac diseases, and hepatitis [6]. At the beginning of the 20th century, in Europe, the hypertensive and antitumor activity of the plant was studied [6]. However, viscum’s therapeutic properties (antidiabetic, analgesic, anti-inflammatory, and hypotensive) were already known in Asian (Israel, China, India and Japan) and African traditional medicine [6,7]. 

Currently, viscum is used as a complementary treatment in cancer therapy in several European countries, where there are different types of commercial phytotherapeutic extracts: Iscador, Isorel, Eurixor, Plenesol, Vysorel, Lektinol, Helixor, Cefalektin, and Lektinol [4,5,6,7]. Some studies report also that its extract enhances an organism’s immune response [4]. However, the phytotherapeutic effects of medicinal plants and their use in the therapy of severe conditions, such as cancer or neurodegenerative diseases, is still a contradictory topic in Western medicine [8,9].

The highly complex chemical composition and biological activity of viscum could be the result of a mixture of biotic (mainly host tree) and abiotic characteristics (water quantity, soil, pH, temperature, sun exposure, harvest season, and growth stage of the plant) [10,11].

Various research reported a large number of phytochemicals, such as lectins (glycopeptides), viscotoxins, terpenoids, coumarins, flavonoids, peptides, carbohydrates, sterols, alkaloids, proteins, amines, polyphenols, amino acids, and lignans [6,7,8,9,10,11,12]. These compounds were studied extensively to establish a relationship between antitumor activity and their biological properties. The main molecules in viscum considered to pose antitumor properties are lectins and viscotoxins. However, recent studies have reported that other secondary metabolites, such as triterpenoids and phenolic derivatives, have anticancer properties [6,7]. It has also been found that the antitumor activity of particular metabolites isolated from viscum is much lower than that of the extract itself. It is due to the synergistic action by which metabolites act [6,7,8,9,10,11,12,13].

Latest studies have shown that viscum extract has positive effects on the quality of life of cancer patients by reducing the side effects of chemo and radiation therapy [14,15,16]. However, the results of the studies indicate that the antitumor activity of viscum seems to be influenced by several factors (the amount of plant used in the extract, the type of host tree, and the harvest period).

Studies have shown the existence of variation in the content of lectins and viscotoxins depending on the season. Thus, there is a maximum of these phytoconstituents in the viscum samples collected in June and December, thus confirming the practices of traditional medicine [17]. Although various recent research reported the cytotoxic, apoptotic, anti-inflammatory, and immunological effects of viscum, the antitumor mechanism is not fully elucidated and not fully understood [15,16].

Gold has influenced human civilization from the humanity dawn through its characteristics and availability, both materially (social hierarchy) and spiritually. From an esoteric and religious point of view, it was considered a symbol of perfection, immortality, rejuvenation, health, and wisdom. In human belief, gold played an essential role: from the symbolism of the sun in astrology to the highest degree of development of matter (mind, spirit, and soul) in alchemy, as well as the renewal and regeneration of humanity to perfection, enlightenment, and spiritual elevation. In Christianity, gold was associated with divine worship and love [18,19].

In medicine, gold has been used for medical purposes since ancient times. In traditional Chinese medicine, gold was used as a therapeutic agent for various ailments (joint, lungs, measles, skin ulcers, wounds, seizures, detoxification, palpitation, coughing, typhoid fever, and so forth) [20]. In Indian traditional medicine (Ayurveda and Siddha), gold is also used for infertility, asthma, diabetes, and cancer [21]. The Ayurvedic gold preparations are a mixture of gold nanoparticles and herbs used, in which the metal had a double role as a carrier and therapeutic agent [22,23].

Since antiquity there has been evidence of the use of gold in traditional European medicine, such as for skin infection treatment and as an antidote to mercury poisoning [24]. Archaeological discoveries show that in ancient Egypt it was used for dental work [25,26]. 

Later, in the Middle Ages, according to the documents of the time, “*aurum potabile*” or other gold-based preparations were used for many types of heart conditions, digestive ailments, baldness, fever, and so on [26,27].

Since the end of the 19th century, gold therapy has been adopted for the treatment of syphilis, tuberculosis, rheumatoid arthritis, and other types of arthritis. Scientific results have shown the ineffectiveness of gold derivatives in treating tuberculosis. It is noteworthy that chrysotherapy (or therapy with gold compounds) is accepted and implemented in modern medicine for rheumatoid arthritis treatment. Gold compounds have demonstrated anti-inflammatory properties and are used as a common medication for chronic and progressive inflammation of bones and joints [26,27,28,29].

Currently, colloidal gold is included in the category of food supplements and is used for antibacterial, antioxidant, and anti-age properties [27,30]. Recent research reported that different gold species possess anticancer, antiproliferative, antimicrobial, anti-inflammatory, antibacterial, anti-rheumatic, and antimalarial properties [27,29,31].

The latest research on the biomedical applications of gold nanoparticles (AuNPs) has shown potential in medical imaging, drug delivery systems, or therapeutic agents for cancer, HIV, neurodegenerative diseases (Alzheimer’s, Parkinson’s), nutrition diseases (diabetes, obesity), ophthalmology, and tissue engineering [28].

AuNPs display versatility, strong surface plasmon absorption, high stability, biocompatibility, and low toxicity in biological environments. Moreover, their high surface-to-volume ratio and predetermined size allow the functionalization of a wide variety of biologically active compounds. Hence, they are especially useful in the design of innovative materials, robust and selective for vectorization, imaging, diagnostics, and cancer therapy [28,31,32,33]. Hereafter, the design of a phyto-engineered-AuNPs carrier assembly will act as a selective vehicle with multi-targeted effects that exert a significant anti-proliferative effect [31,32,33,34].

There is relatively little research on Romanian viscum aimed mainly at determining certain constituents such as polyphenols, total proteins, amino acids, peptides, lectins, and viscotoxins from various parts of the plant (young leaves and branches). Moreover, most of these studies used viscum collected from *Acer campestre, Mallus domestica, Fraxinus excelsior, Populus nigra*, and *R. pseudoacacia* [5,35,36,37,38,39,40].

To our best knowledge, this study investigates for the first time the complete low-molecular-weight metabolite profile of the Romanian *Viscum album* hosted on *Quercus robur* L. (collected on summer solstice). Furthermore, our previous studies were focused on identifying the amino acids and thionines in viscum (collected near the winter solstice) from the same oak species [5].

The results of this study confirm, for the first time, the existence of a difference between the profile of amino acids and small peptides in the viscum samples grown on the same Romanian oak species and collected in the two periods considered critical by traditional European medicine.

Another novelty of this study is that, for the first time, an active, selective, and specific target-engineered carrier assembly with AuNPs features, which collectively capitalizes the therapeutic properties of Romanian viscum (whole plant), was developed and analyzed.

## 2. Results and Discussion 

Plants, and medicinal plants in particular, contain a remarkable complex mixture of natural compounds with unique chemical structures and a significant number of stereogenic centers. A herb’s biological activity is due to the synergistic interaction of all its compounds within an organism. Therefore, the pharmacological action of a plant extract is attributed to a whole multi-component mixture of constituent natural compounds [41,42,43].

On the other hand, an important aspect that must be considered for herb chemical screening, is that its metabolites profile varies depending on various endogenous or ecological factors [38,44,45].

Numerous research studies have focused on the highly complex phytochemical content of viscum. However, the correlation between their metabolic profiles and bioactivity has not yet been established. One of the main reasons is the variation in the composition of the phytoconstituents depending on the host tree [39,46,47].

A qualitative untargeted metabolomics profiling strategy based on a mass spectrometry approach is a powerful tool for fast and cost-effective chemical screening of secondary metabolites from natural compounds. 

**Figure 1:**
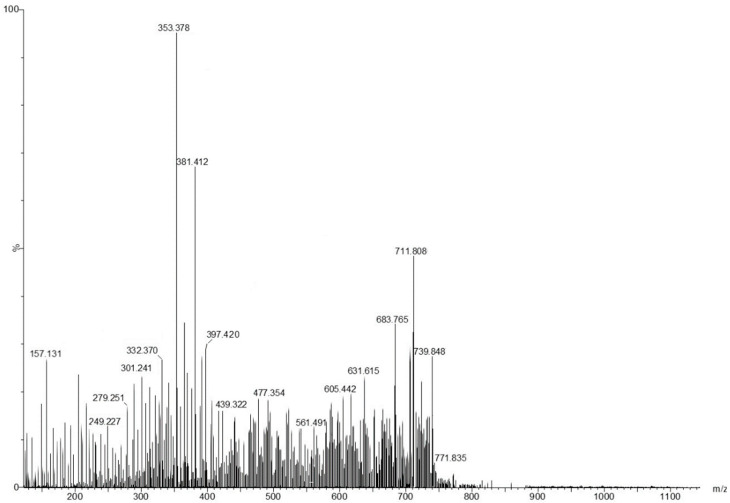
The mass spectrum of Romanian *Viscum album* hosted on *Quercus robur* L. (collected on summer solstice).

The chemical screening profile of low-molecular-weight metabolites from viscum was tentatively identified through electrospray ionization–quadrupole time-of-flight mass spectrometry (ESI-QTOF-MS) analysis. The mass spectra of the components identified were accomplished by connecting with those of NIST/EPA/NIH, Mass Spectral Library 2.0 database and reviewing the literature [46,47,48,49,50,51]. The mass spectrum and the components identified by ESI-QTOF-MS analysis are presented in Figure 1 and Table 1.

### 2.1. Screening and Classification of the Differential Metabolites

A total of 140 compounds were found and identified by mass spectroscopy analysis, including primary metabolites (amino acids, peptides, organic acids, nucleosides, etc.) and secondary metabolites (terpenoids, flavonoids, sterols, coumarins, alkaloids, phenolic acids, sterols, fatty acids, and miscellaneous), confirming the results reported in other studies on this plant [10,11,40,41,42,43,44,45,46,47,48,49,50,51,52,53,54,55,56,57,58,59]. These phytochemicals were assigned to different chemical categories: flavonoids (22.14%), amino acids and peptides (20%), terpenoids (17.85%), phenolic acids (7.85%), fatty acids (7.85%), organic acids (4.28%), nucleosides (3.57%), alcohols and esters (3.57%), coumarins (2.14%), alkaloids (2.14%), amines (2.14%), lignals (2.14%), sterols (1.42%), aldehydes and ketones (1.42%), and other. Flavonoids, amino acids and peptides, and terpenoids represent about 60% of all metabolites identified in *Viscum album*. The distribution of the identified metabolites in various chemical classes is listed in Table 2.

**Figure 2:**
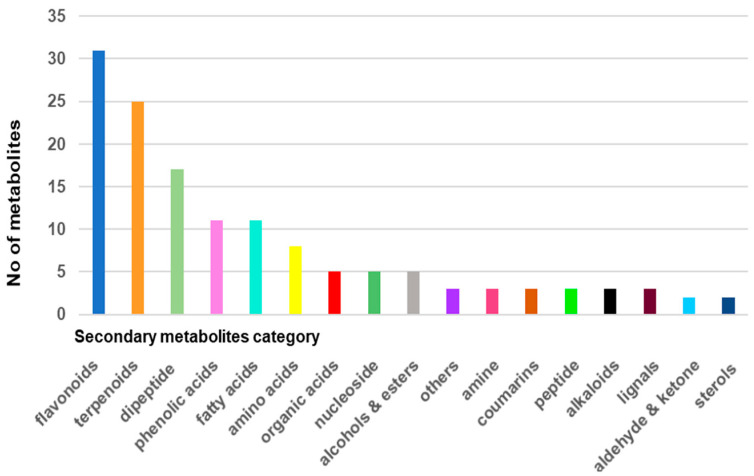
Metabolite classification bar chart of Romanian *Viscum album* hosted on *Quercus robur* L. (collected on summer solstice).

Figure 2 presents the metabolite classification chart and was obtained on the basis of the data analysis reported in Table 2. 

Amino acids and peptides: a total of 28 compounds were identified in the plant extract. A large part of these compounds (over 80%) is essential amino acids (phenylalanine, leucine, tryptophan, valine, methionine, histidine, and arginine). The non-essential amino acids (tyrosine, glutamic acid, cysteine, proline, glycine, alanine, and asparagine) are present only in a smaller proportion, about 20% of them [60,61,62,63,64]. The majority of the amino acids identified in the viscum sample (arginine, phenylalanine, tryptophan, histidine, glutamic acid, methionine, glycine, and proline) exhibit cytotoxicity, antiproliferative, and immunomodulant activity [61,62,63,64,65].

Several new small peptides (17 dipeptides, a cyclic peptide, and one tripeptide) have been identified, indicating that the profile of small peptides varies in the chemical composition of the viscum grown on the same oak species during the summer solstice and winter solstice [5]. This difference in the composition of small peptides in the viscum samples (on oak in the same geographical region) confirms the variation in plant therapeutic properties (especially antitumor and immunomodulatory) depending on the two periods considered essential in traditional European medicine [7,17].

Terpenoids and sesquiterpenes are among the main categories of metabolites found in viscum samples. Extensive studies report their antimicrobial, antioxidant, anticonvulsive, analgesic, neuroprotective, anti-inflammatory, anti-allergic, and antitumoral activities [51,60,65,66,67,68].

Coumarins are another class of phytoconstituents with high biological activity: antiviral, antimicrobial, antioxidant, analgesic, anticancer, anti-inflammatory, and anti-neurodegenerative [60,69]. Studies have shown coumarins’ therapeutic applications in several types of cancer: leukemia, breast, renal, prostate, and malignant melanoma [65,70].

Flavonoids are the largest class of metabolites (over 30 different compounds) in the viscum sample. These biomolecules have numerous therapeutic effects: antioxidant, antitumoral, cardiovascular system protection (atherosclerosis, antiplatelet), antimicrobial, anti-inflammatory, and neurodegenerative diseases (Alzheimer) [60,65,71,72,73].

Phenolic acids are reported to act as an antioxidant, anti-inflammatory, antitumoral, neuroprotective, antimicrobial, and antidiabetic agents [60,65,74,75].

Sterol and steroids have been demonstrated to have anti-inflammatory, antitumoral, antidiabetic, antioxidant, anti-atherosclerotic, neuroprotective, immunomodulatory, osteoporosis protection, and cardiovascular protective (anti-atherosclerosis, anti-hemolytic) activities [60,65,76].

Fatty acids represent another important class of secondary metabolites, representing approximately 8% of the total metabolites identified in the viscum sample. Various studies highlight their importance for human health, such as anti-inflammatory, antioxidant, neuroprotective (stroke, Alzheimer’s disease), and cardiovascular protective activity (anti-arrhythmic, dyslipidemia, hypertension, and anti-thrombotic) [60,65,77].

Carbohydrates and polysaccharides are biomolecules involved in the different beneficial roles for human health: anti-inflammatory, antiviral (anti-HIV), antioxidant (anti-ageing), digestive protection, cardio-protective, anti-arthritic, antibacterial, immunomodulatory, anti-diabetic, and anti-tumoral [60,65,78,79].

Glycosides are plant metabolites that act as antitumoral agents, especially for gastric cancer and chronic granulocytic leukemia [65,79].

Miscellaneous compounds, for instance, nonalide (lactone), identified in the viscum sample extract have antifungal activity, antimalarial, and cytotoxic activities [51].

### 2.2. Engineered Viscum–AuNPs Carrier Assembly 

Development of targeted assembly rides on a robust and complex carrier assembly design that collectively combines the therapeutic properties of both components (viscum and an inorganic component) and the physicochemical characteristics of AuNPs (surface plasmon resonance, high surface area, conductivity, and low toxicity) [80,81,82]. 

Active targeting of an engineered carrier assembly relies upon a tailored surface to ensure high selectivity, vectorization, and specificity, and thus exert a significant therapeutic effect [80,81,82,83,84,85]. 

Development of targeted assembly rides on the construction of a robust, complex carried assembly that combines collectively the structural features of AuNPs (nontoxic, high biocompatibility and solubility, non-immunogenicity, and distinctive optical properties) with remarkable antitumor effects of both components (viscum and inorganic component) [28,34,80]. 

### 2.3. FT-IR Spectroscopy 

**Figure 3:**
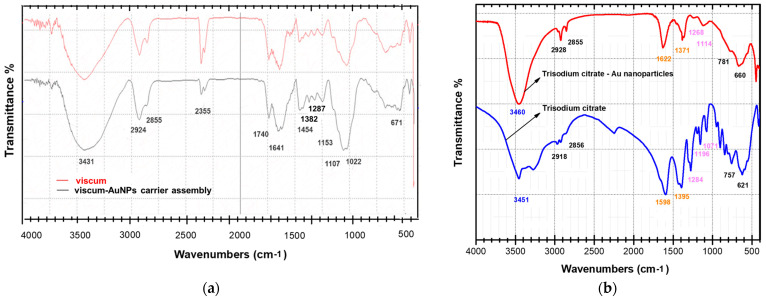
(**a**) FT-IR spectrum of the viscum sample and viscum–AuNPs carrier assembly; (**b**) FT-IR spectrum of AuNPs coated with trisodium citrate and the trisodium citrate spectrum.

The preparation of an engineered viscum–AuNPs carrier assembly was investigated by FT-IR spectroscopy to identify the functional groups specific to functional groups specific its two components, viscum and AuNPs. The individual FT-IR spectrum of the viscum and carrier assembly is shown in Figure 3a,b. The FT-IR absorption bands identified in the viscum sample are presented in Table 3.

The FTIR peak of AuNPs coated with trisodium citrate (surfactant) (Figure 3b) presents the vibrational bands characteristic of a surfactant: at 3460 cm^−1^ (associated with a H–OH stretching vibration) and 2918 cm^−1^; at 2856 cm^−1^ (attributed with CH- asymmetric and symmetric stretching vibrations); at 1598 cm^−1^ (associated with a COO- stretching vibration); at 1395 cm^−1^; and at 757 cm^−1^ (assigned to C–H bending). 

The formation of an engineered viscum–AuNPs carrier assembly was successfully achieved and was confirmed through FT-IR spectroscopy. The spectra of nanoparticle carrier assembly (Figure 3a) show the characteristic absorption bands of the viscum sample as well as the AuNPs coated with a surfactant (trisodium citrate) (Figure 3a,b). In addition, the peaks at 1622, 1371, 1258, 1114, and 660 cm^−1^ were found in the synthesized AuNP solution (Figure 3a) and are shifted to higher wavenumbers (1641, 1382, 1267, 1153 and 671 cm^−1^), indicating binding of nanoparticles to the functional groups O–H, C=O, C–N, C–S, and C–O belonging to the various phytoconstituents (Figure 3a,b and Table 3) [79,80,81,82,83,84,85,86,87,88,89,90,91]. In particular, the hydroxyl group is considered to be involved in the binding of AuNPs [85,86,87,88,89,90,91,92,93,94,95,96,97,98].

### 2.4. X-ray Diffraction Spectroscopy

**Figure 4:**
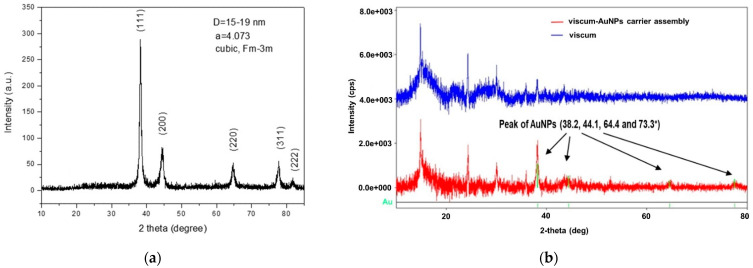
(**a**) Powder XRD patterns of the AuNPs; (**b**) XRD patterns of the viscum sample and engineered viscum–AuNPs carrier assembly.

Figure 4a,b present the XRD patterns of the AuNPs, viscum sample, and the engineered viscum–gold nanoparticles carrier assembly.

Figure 4a shows the specific XRD spectrum of AuNPs. The mean diameter (D) of the gold crystallites, calculated by using the Debye–Scherrer formula, was less than 20 nm. The AuNPs were well crystalized, with well-defined peaks.

The diffraction pattern of the viscum (Figure 4b) is in the range of 12–45°, with large bands and weak peaks characteristic of amorphous phases that can be attributed to plant fibers and minerals in the form of hydroxide.

The pattern of the engineered viscum–AuNPs carrier assembly has a shape similar to that of the viscum. In this spectrum, besides the wide bands and the weak peaks of the plant, one can observe, but much attenuated, the peaks of the AuNPs at 38.2, 44.1, 64.4, and 77.3°.

### 2.5. Scanning Electron Microscopy (SEM) 

**Figure 5:**
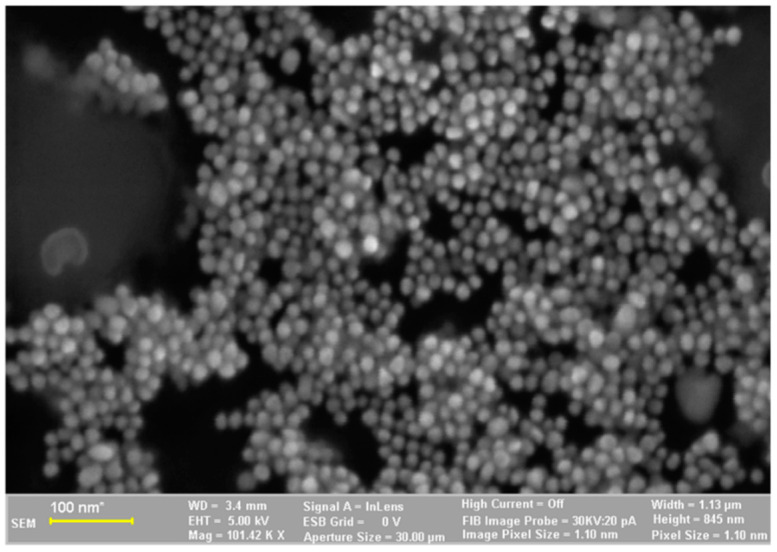
Two-dimensional image of the AuNPs obtained by the SEM technique.

**Figure 6:**
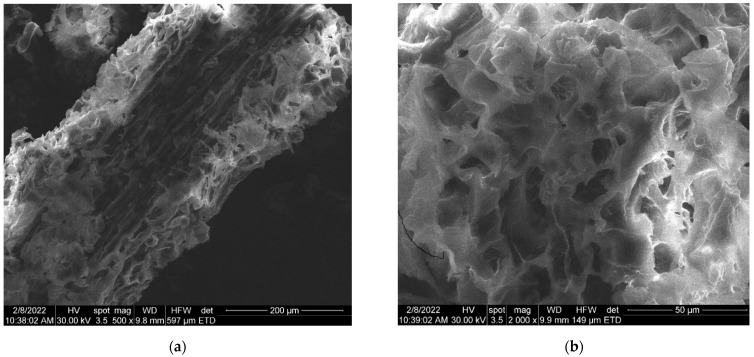
(**a**) Two-dimensional image of the viscum sample obtained by the SEM technique (magnitude 200 µm). (**b**) Two-dimensional image of the viscum sample obtained by the SEM technique (magnitude 50 µm).

**Figure 7:**
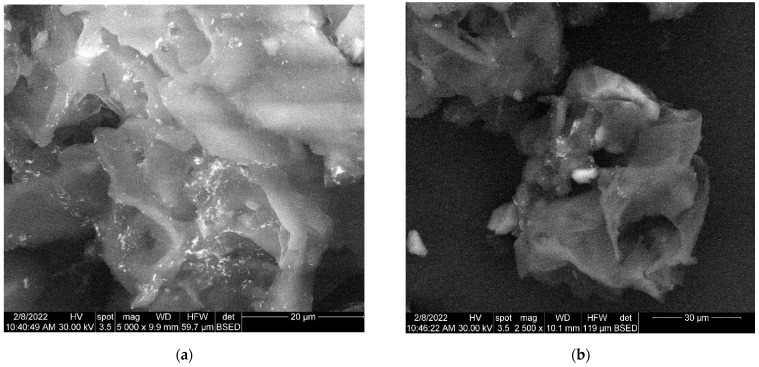
(**a**) Two-dimensional image of the engineered viscum–AuNPs carrier assembly obtained by the SEM technique (magnitude 20 µm). (**b**) Two-dimensional image of the engineered viscum–AuNPs carrier assembly obtained by the SEM technique (magnitude 30 µm).

**Figure 8:**
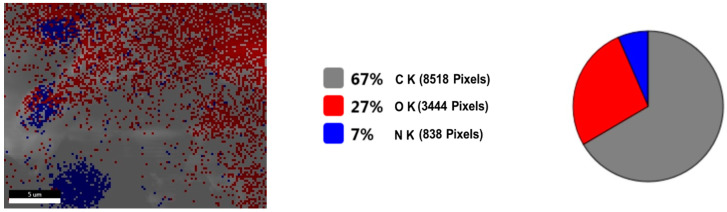
*Viscum* sample: SEM—live map.

**Figure 9:**
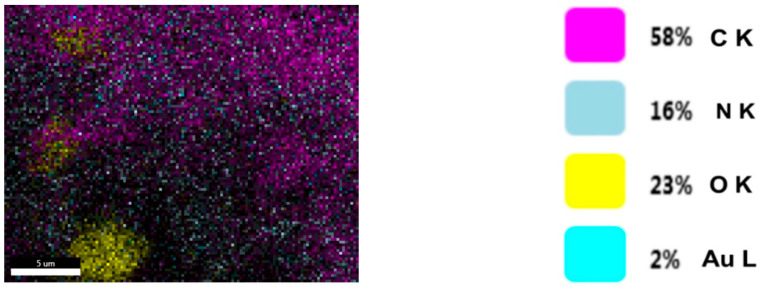
Engineered viscum–AuNPs carrier assembly: SEM—live map.

The SEM images of the synthesized AuNP solution, viscum sample, and engineered herb–AuNPs carrier assembly are presented in Figure 5, Figure 6, Figure 7, Figure 8 and Figure 9. SEM micrographs of the AuNPs (Figure 5a) indicate a surface structure with agglomerated regions of regular, spherical particles, with dimensions between 8 and 18 nm [99]. The morphology of the viscum sample (Figure 6a,b) displays a fibrous structure surface, with irregular, porous areas with dimensions less than 1 mm. The presence of pores suggests an easy fixation of AuNPs on the surface of the plant sample.

The morphology of engineered viscum–AuNPs carrier assembly indicates the presence of AuNPs and agglomerations of AuNPs, fixed on the surface of viscum particles (Figure 7a) as well as loaded into the pores of viscum particles (Figure 7b).

Figure 8 shows the live map of viscum and the distribution of the identified elements. Figure 9 shows the live map for the engineered viscum–AuNPs carrier assembly and the distribution of the identified elements. The comparative analysis of Figure 8 shows the live map for viscum and engineered viscum–AuNPs carrier assembly (Figure 9) and highlights the presence of differences regarding the proportion of identification elements in the two samples, due to the formation of the viscum–metal carrier assembly. SEM analysis and live map confirms the obtaining of the engineered viscum–AuNPs carrier assembly [74].

### 2.6. UV–VIS Spectroscopy

The optical properties (surface plasmon resonance) of AuNPs and the achievement of an engineered viscum–AuNPs carrier assembly were monitored through UV–Vis spectroscopy.

**Figure 10:**
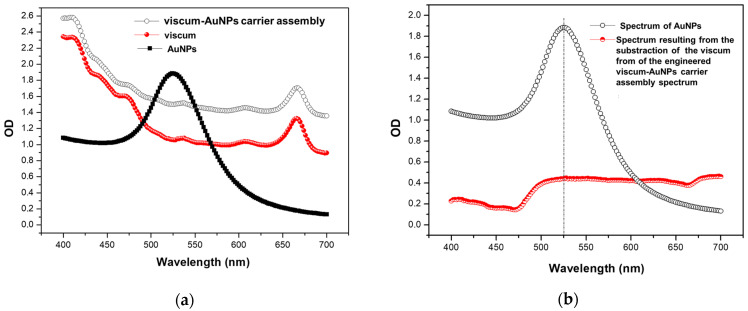
(**a**) UV–Vis spectra of the AuNPs, viscum, and engineered viscum–AuNPs carrier assembly respectively. (**b**) UV–Vis spectrum resulting from subtracting the UV–VIS spectra of the AuNPs from the UV–Vis spectrum of the engineered viscum–AuNPs carrier assembly.

In Figure 10a, it can be seen that AuNPs show a peak absorption at 526 nm, but this absorption band is not visible in the case of the engineered viscum–AuNPs carrier assembly. The viscum absorption spectra and the final material (engineered viscum–AuNPs carrier assembly) do not show significant differences. Moreover, the specific absorption peak of the AuNPs is not visible in the spectrum of viscum–AuNPs. However, there is a clear difference in the absorbance intensity between the first two spectra, starting at about 475 nm. This was better emphasized by subtracting the viscum spectrum from the spectrum of the engineered viscum–AuNPs carrier assembly (Figure 10b). After subtraction, the surface plasmon band (at 526 nm) in the spectrum of AuNPs is now discreetly observed also in the subtraction curve, even if the profiles of the curves match approximately up to 526 nm only. This demonstrates that viscum successfully incorporated the AuNPs and that the engineered viscum–AuNPs carrier assembly was obtained.

## 3. Materials and Methods

All used reagents were analytical grade. Methanol and chloroform were purchased from VWR (Wien, Austria) and used without further purification.

### 3.1. Carrier Assembly Components Preparation

#### AuNPs Synthesis

The citrate synthesis of gold nanoparticles was achieved by the following procedure: in conical flasks (2000 mL), 0.5 g AuCl_4_H was weighted and a constant volume (1450 mL) of ultrapure water was added under magnetic stirring (800 rpm). Then, 50 mL of sodium citrate solution (1.6%) was added rapidly. The mixture was kept at room temperature (22 °C) for 40 min and 800 rpm. 

### 3.2. Plant Sample Preparation

The five individual viscum (*Viscum album L*) samples (whole plant) were collected from three trees (*Quercus robur* L.) in June 2021 from the area of Timis county, Romania (geographic coordinates 45°47′5″ N 21°16′0″ E), and were taxonomically authenticated at West University of Timisoara, Romania. The plant samples were rapidly frozen in liquid nitrogen (190 °C), ground and sieved to obtain a particle size lower than 0.5 mm, and kept at −40 °C to avoid enzymatic conversion or metabolite degradation. 

### 3.3. Plant Preparation for Chemical Screening

For each analysis, 1.5 g of dried viscum sample was subject to sonication extraction in 25 mL of solvent (methanol/chloroform = 1:1) for 30 min at 38 °C with a frequency of 60 kHz. The solution was concentrated using a rotavapor and the residue was dissolved in MeOH. The extract was centrifuged, and the supernatant was filtered through a 0.2 µm syringe filter and stored at –18 °C until analysis until MS analysis. All samples were prepared in triplicate.

### 3.4. Mass Spectrometry

The experiments were conducted using EIS-QTOF-MS from Bruker Daltonics, Bremen, Germany. All mass spectra were acquired in the positive ion mode within a mass range of 100–3000 m/z, with a scan speed of 2.1 scans/s. The source block temperature was kept at 80 °C. The reference provided a spectrum in positive ion mode with fair ionic coverage of the m/z range scanned in full-scan MS. The resulting spectrum was a sum of scans over the total ion current (TIC) acquired at 25–85 eV collision energy to provide the full set of diagnostic fragment ions. 

The metabolites were identified via comparison of their mass spectra with those of the standard library NIST/NBS-3 (National Institute of Standards and Technology/National Bureau of Standards) spectral database and the identified phytoconstituents are presented in Table 1.

### 3.5. Phyto-Engineered AuNPs Carrier Assembly Preparation

For each analysis, 2.5 g of sample was prepared from dried viscum (whole plant, ground and sieved to obtain a particle size lower than 0.5 mm) and a AuNPs solution was added (viscum/AuNPs nanoparticles = 1:4) at room temperature (22 °C) and under magnetic stirring (400 rpm) for 24 h. The obtained mixture was filtered (Φ185 mm filter paper) and then dried in oven at 40 °C for 4 h.

### 3.6. Characterisation of the Engineered Viscum–AuNPs Carrier Assembly 

#### 3.6.1. UV–Vis Analysis

The measurements were conducted using a spectrophotometer UV–VIS Perkin-Elmer Lambda 35 packing pre-aligned halogen and deuterium lamps. The two sources of radiation cover the range of wavelengths of 190–1100 nm and a variable bandwidth range of 0.5 to 4 nm.

#### 3.6.2. Fourier Transform Infrared (FTIR) Spectroscopy

FTIR spectra were obtained by using the KBr pellet method ranging from 4000 cm^−1^ to 400 cm^−1^, with a Perkin-Elmer Spectrum 100 FT-IR (Perkin–Elmer, Waltham, MA, USA).

The X-ray powder diffraction (XRD) pattern was performed using a Rigaku Ultima IV diffractometer equipped with a D/teX ultra-detector and operating at 40 kV and 40 mA, with monochromatic CuKα radiation (λ = 1.5406Å), in the 2θ range 10–80°, with a scan speed of 5°/min and a step size of 0.01°. The XRD patterns were compared with those from the ICDD Powder Diffraction Database, (ICDD file 04-015-9120). The average crystallite size and the phase content was calculated using the whole pattern profile fitting method (WPPF).

Scanning electron microscopy (SEM) micrographs were obtained with an SEM-EDS system (QUANTA INSPECT F50) equipped with a field-emission gun (FEG), 1.2 nm resolution, and energy dispersive X-ray spectrometer (EDS) with an MnK resolution of 133 eV.

## 4. Conclusions

In this study, the low molecular mass metabolites profiling of *Viscum album* (growing wild in Romania and hosted on *Quercus robur* L.) was accomplished. The biological activities were discussed for each metabolite category. Compared to our previous studies, 19 new small peptides were identified, which indicates that the profile of small peptides varies in the chemical composition of the viscum grown on the same oak species during the summer solstice and winter solstice.

Furthermore, a new and specific target-engineered viscum–AuNPs carrier assembly, with unique optical properties and great potential to increase the effectiveness of antitumor action and reduce unwanted side effects, was developed. The complete morpho-structural characterization of the viscum–AuNPs carrier assembly was performed. Further research is necessary to investigate the biological activity and biocompatibility of viscum extracts and the newly engineered viscum–AuNPs carrier assembly.

## Figures and Tables

**Table 1:** Components identified through electrospray ionization–quadrupole time-of-flight mass spectrometry (ESI-QTOF-MS) analysis.

Compound No.	m/z Detected	Theoretic m/z	Formula	Tentative of Identification	Category	Ref.
1	104.16	104.17	C_5_H_14_NO^+^	choline	amine	[48]
2	111.15	111.15	C_5_H_9_N_3_	histamine	amine	[48]
3	121.14	121.16	C_3_H_7_NO_2_S	cysteine	amino acids	[9]
4	122.15	122.16	C_8_H_10_O	2-phenylethanol	alcohol	[50]
5	128.22	128.21	C_8_H_16_O	1-octene-3-ol	alcohol	[51]
6	131.16	131.17	C_6_H_13_NO_2_	leucine	amino acids	[52]
7	136.22	136.23	C_10_H_16_	sabinene	terpenoids	[51]
8	138.11	138.12	C_7_H_6_O_3_	salicylic acid	phenolic acid	[10,40]
9	144.20	144.21	C_8_H_16_O_2_	octanoic acid	fatty acids	[51]
10	146.13	146.14	C_9_H_6_O_2_	coumarin	coumarins	[49]
11	146.22	146.21	C_7_H_16_NO_2_^+^	acetylcholine	amine	[48]
12	147.12	147.13	C_5_H_9_NO_4_	glutamic acid	amino acids	[11,52]
13	148.15	148.16	C_9_H_8_O_2_	cinamic acid	phenolic acid	[40]
14	149.22	149.21	C_5_H_11_NO_2_S	methionine	amino acids	[52]
15	150.21	150.22	C_10_H_14_O	safranal	terpenoids	[51]
16	152.14	152.15	C_8_H_8_O_3_	vanillin	aldehydes, phenols	[50]
17	152.21	152.23	C_10_H_16_O	citral	terpenoids	[51]
18	154.11	154.12	C_7_H_6_O_4_	gentisic acid	phenolic acid	[10,40]
19	154.26	154.25	C_10_H_18_O	geraniol	terpenoids	[51]
20	155.14	155.15	C_6_H_9_N_3_O_2_	histidine	amino acids	[52]
21	156.21	156.22	C_9_H_16_O_2_	nonanolide	lactone	[51]
22	162.17	162.18	C_10_H_10_O_2_	cinnamic acid methyl ester	ester	[50]
23	164.14	164.16	C_9_H_8_O_3_	p-coumaric acid	phenolic acid	[40,49]
24	166.18	166.17	C_9_H_10_O_3_	tropic acid	organic acid	[52]
25	170.11	170.12	C_7_H_6_O_5_	gallic acid	phenolic acid	[52]
26	174.10	174.11	C_6_H_6_O_6_	aconic acid	miscellaneous	[52]
27	174.19	174.2	C_6_H_14_N_4_O_2_	arginine	amino acids	[11,48,52]
28	176.11	176.12	C_6_H_8_O_6_	ascorbic acid	organic acid	[40,49]
29	176.16	176.17	C_10_H_8_O_3_	6-hydroxy-4-methylcoumarin	coumarins	[52]
30	180.15	180.16	C_9_H_8_O_4_	caffeic acid	phenolic acid	[40,52,53]
31	181.18	181.19	C_9_H_11_NO_3_	tyrosine	amino acids	[11,52]
32	182.16	182.17	C_9_H_10_O_4_	veratric acid	phenolic acid	[10]
34	186.11	186.12	C_8_H_14_N_2_O_3_	prolyl-alanine	dipeptide	[52]
35	188.21	188.22	C_8_H_16_N_2_O_3_	leucyl-glycine	dipeptide	[52]
36	192.11	192.12	C_6_H_8_O_7_	citric acid	organic acid	[52]
37	193.16	192.17	C_7_H_12_O_6_	quinic acid	organic acid	[49,53,54]
38	192.29	192.3	C_13_H_20_O	ionone	ketone	[51]
39	194.17	194.18	C_10_H_10_O_4_	ferulic acid	phenolic acid	[10,40]
40	194.30	194.31	C_13_H_22_O	geranylacetone	terpenoids	[51]
41	196.23	196.24	C_11_H_16_O_3_	loliolide	terpenoid	[52]
42	196.27	196.29	C_12_H_20_O_2_	nerol acetate	terpenoids	[51]
43	198.16	198.17	C_9_H_10_O_5_	syringic acid	phenolic acid	[40,53]
44	202.23	202.25	C_9_H_18_N_2_O_3_	leucyl-alanine	dipeptide	[52]
45	204.21	204.22	C_11_H_12_N_2_O_2_	tryptophan	amino acids	[52]
46	204.34	204.35	C_15_H_24_	caryophyllene	terpenoids	[51]
47	204.36	206.37	C_15_H_26_	cadinene	terpenoids	[51,52]
48	208.19	208.21	C_8_H_16_O_6_	viscumitol	saccharides	[49]
49	216.27	216.28	C_10_H_20_N_2_O_3_	valylvaline	dipeptide	[52]
50	222.35	222.37	C_15_H_26_O	cadinol	terpenoids	[51,52]
51	224.19	224.21	C_11_H_12_O_5_	sinapic acid	phenolic acid	[40,53]
52	224.29	224.3	C_13_H_20_O_3_	vomifoliol	terpenoids	[52]
53	226.21	226.23	C_11_H_14_O_5_	genipin	terpenoids	[52]
54	228.28	228.29	C_11_H_20_N_2_O_3_	prolyl-leucine	dipeptide	[52]
55	229.22	229.23	C_9_H_15_N_3_O_4_	asparaginyl-proline	dipeptide	[52]
56	230.29	230.3	C_15_H_18_O_2_	dehydrocostuslactone	terpenoids	[52]
57	234.19	234.2	C_12_H_10_O_5_	7-methoxycoumarin-4-acetic acid	coumarins	[52]
58	234.37	234.38	C_15_H_26_N_2_	sparteine	alkaloid	[55]
59	236.26	236.27	C_12_H_16_N_2_O_3_	phenylalanylalanine	dipeptide	[52]
60	236.34	236.35	C_15_H_24_O_2_	curcumol	terpenoids	[52]
61	240.49	240.5	C_17_H_36_	heptadecane	hydrocarbons, lipids	[51]
62	242.21	242.23	C_10_H_14_N_2_O_5_	thymidine	nucleoside	[40,49]
63	242.39	242.4	C_15_H_30_O_2_	pentadecanoic acid	fatty acids	[51]
64	243.21	243.22	C_9_H_13_N_3_O_5_	cytidine	nucleoside	[52]
65	244.19	244.2	C_9_H_12_N_2_O_6_	uridine	nucleoside	[52]
66	244.23	244.24	C_10_H_16_N_2_O_5_	prolylglutamic acid	dipeptide	[52]
67	244.31	244.33	C_12_H_24_N_2_O_3_	isoleucyl-isoleucine	dipeptide	[11,52]
68	248.35	248.36	C_15_H_24_N_2_O	lupanine	alkaloid	[55]
69	250.26	250.27	C_8_H_14_N_2_O_5_S	gamma-glutamylcysteine	dipeptide	[49]
70	250.37	250.38	C_15_H_26_N_2_O	retamine	alkaloid	[53]
71	256.41	256.42	C_16_H_32_O_2_	palmitic acid	fatty acids	[52]
72	262.29	262.3	C_14_H_18_N_2_O_3_	prolylphenylalanine	dipeptide	[52]
74	264.31	264.32	C_14_H_20_N_2_O_3_	phenylalanylvaline	dipeptide	[52]
74	267.23	267.24	C_10_H_13_N_5_O_4_	adenosine	nucleoside	[40,52]
75	272.24	272.25	C_15_H_12_O_5_	naringenin	flavonoids	[40,54,55]
76	278.29	278.3	C_14_H_18_N_2_O_4_	tyrosyl-L-proline	dipeptide	[55]
77	278.39	278.4	C_18_H_30_O_2_	linolenic acid	fatty acids	[46]
78	278.33	278.35	C_15_H_22_N_2_O_3_	leucyl-phenylalanine	dipeptide	[52]
79	280.39	280.4	C_18_H_32_O_2_	linoleic acid	fatty acids	[52]
80	283.21	283.24	C_10_H_13_N_5_O_5_	guanosine	nucleoside	[52]
81	284.49	284.5	C_18_H_36_O_2_	stearic acid	fatty acids	[52]
82	286.23	286.24	C_15_H_10_O_6_	luteolin	flavonoids	[52]
83	288.26	288.25	C_15_H_12_O_6_	eriodictyol	flavonoids	[49]
84	292.39	292.4	C_18_H_28_O_3_	9-OxoOTrE	fatty acids	[52]
85	292.34	294.35	C_15_H_22_N_2_O_4_	tyrosylleucine	dipeptide	[52]
86	296.49	296.5	C_20_H_40_O	phytol	terpenoids	[51]
87	298.39	298.4	C_19_H_22_O_3_	acerogenin G	flavonoids	[46]
88	300.25	300.26	C_16_H_12_O_6_	rhamnocitrin	flavonoids	[49]
89	302.22	302.23	C_15_H_10_O_7_	quercetin	flavonoids	[40,53,54]
90	302.27	302.28	C_16_H_14_O_6_	homoeriodictyol	flavonoids	[46]
91	300.39	300.4	C_19_H_24_O_3_	centrolobol	flavonoids	[46]
92	307.31	307.33	C_10_H_17_N_3_O_6_S	glutathione	peptides	[48,49,52]
93	308.49	308.5	C_20_H_36_O_2_	terpineol	terpenoids	[51]
94	312.39	312.4	C_18_H_20_N_2_O_3_	phenylalanylphenylalanine	dipeptide	[52]
95	312.49	312.5	C_20_H_40_O_2_	arachidic acid	fatty acids	[52]
96	314.27	314.29	C_17_H_14_O_6_	ermanin	flavonoids	[52]
97	314.49	314.5	C_18_H_34_O_4_	12,13-DiHOME	fatty acids	[52]
98	316.25	316.26	C_16_H_12_O_7_	rhamnetin	flavonoids	[52]
99	317.39	317.4	C_17_H_23_N_3_O_3_	leucyl-tryptophan	dipeptide	[52]
100	328.39	328.4	C_19_H_20_O_5_	hirsutanone	diarylheptanoids	[49,52]
101	330.27	330.29	C_17_H_14_O_7_	rhamnazin	flavonoids	[49]
102	341.39	341.4	C_17_H_31_N_3_O_4_	Ile-Pro-Ile	peptides	[52]
103	342.33	342.34	C_16_H_22_O_8_	coniferin	glucoside	[56]
104	344.39	344.3	C_18_H_16_O_7_	santin	flavonoids	[52]
105	354.29	354.31	C_16_H_18_O_9_	chlorogenic acid	phenolic acid	[40,46,49,54]
106	360.29	360.3	C_18_H_16_O_8_	rosmarinic acid	phenolic acid	[10,40]
107	368.59	368.6	C_24_H_48_O_2_	lignoceric acid	fatty acids	[52]
108	372.39	372.4	C_17_H_24_O_9_	syringin	flavonoids	[11,52]
109	386.39	386.4	C_19_H_30_O_8_	roseoside	terpenoids	[52]
110	388.39	388.4	C_21_H_24_O_7_	medioresinol	lignal	[52]
111	399.69	399.7	C_26_H_52_O_2_	cerotic acid	fatty acids	[52]
112	406.39	406.4	C_20_H_22_O_9_	viscutin-3	flavonoids	[49]
113	412.69	412.7	C_30_H_52_	lupane	terpenoids	[49]
114	414.69	414.7	C_29_H_50_O	β-sitosterol	sterols	[49,57]
115	418.39	418.4	C_22_H_26_O_8_	syringaresinol	lignal	[49]
116	426.69	426.7	C_30_H_50_O	lupeol	terpenoids	[46,49]
117	434.29	434.3	C_20_H_18_O_11_	avicularin	flavonoids	[55]
118	434.39	434.4	C_21_H_22_O_10_	naringenin-7-O-glucoside	flavonoids	[54]
119	442.39	442.4	C_20_H_26_O_11_	visartiside D	flavonoids	[54]
120	442.69	442.7	C_30_H_50_O_2_	betulin	terpenoids	[46]
121	448.39	448.4	C_21_H_20_O_11_	quercitrin	flavonoids	[49]
122	450.39	450.4	C_21_H_22_O_11_	eriodictyol-7-O-glucoside	flavonoids	[50]
123	456.69	456.7	C_30_H_48_O_3_	betulinic acid	terpenoids	[46,57]
124	464.39	464.4	C_21_H_20_O_12_	hyperoside	flavonoids	[51]
125	468.79	468.8	C_32_H_52_O_2_	β-amyrin acetate	terpenoids	[50]
126	476.41	476.43	C_23_H_24_O_11_	flavoyadorinin B	flavonoids	[49]
127	478.39	478.4	C_22_H_22_O_12_	isorhamnetin-3-O-rutinoside	flavonoids	[52,53,56]
128	492.39	492.4	C_23_H_24_O_12_	flavoyadorinin A	flavonoids	[49]
129	526.49	526.5	C_27_H_26_O_11_	viscutin-1	flavonoids	[49]
130	532.49	532.5	C_27_H_32_O_11_	visartiside E	flavonoids	[50]
131	536.89	536.9	C_40_H_56_	carotene	miscellaneous	[48]
132	565.79	565.8	C_30_H_55_N_5_O_5_	viscumamide	peptides	[58,59]
133	568.49	568.5	C_29_H_28_O_12_	viscutin-2	flavonoids	[49]
134	576.79	576.8	C_35_H_60_O_6_	daucosterol	sterols	[52]
135	596.49	596.5	C_27_H_32_O_15_	viscumneosides I	flavonoids	[49]
136	608.49	608.5	C_28_H_32_O_15_	homoflavoyadorinin B	flavonoids	[52]
137	636.59	636.6	C_29_H_32_O_16_	viscumneoside IV	flavonoids	[49]
138	728.59	728.6	C_32_H_40_O_19_	viscumneoside V	flavonoids	[49]
139	768.69	768.7	C_34_H_40_O_20_	viscumneoside VII	flavonoids	[49]
140	784.96	784.97	C_41_H_68_O_14_	astragaloside IV	terpenoid	[49]

**Table 2:** Classification of metabolites from the *Viscum album* sample on different chemical categories.

Chemical Class	Metabolite Name
Flavonoids	naringenin
	acerogenin G
	luteolin
	eriodictyol
	rhamnocitrin
	centrolobol
	quercetin
	homoeriodictyol
	ermanin
	rhamnetin
	rhamnazin
	santin
	syringin
	viscutin-3
	avicularin
	naringenin-7-O-glucoside
	visartiside D
	quercitrin
	eriodictyol-7-O-glucoside
	hyperoside
	flavoyadorinin B
	isorhamnetin-3-O-rutinoside
	flavoyadorinin A
	viscutin-1
	visartiside E
	viscutin-2
	viscumneosides I
	homoflavoyadorinin B
	viscumneoside IV
	viscumneoside V
Amino acids and peptides	cysteine
	leucine
	glutamic acid
	methionine
	histidine
	arginine
	tyrosine
	prolyl-alanine
	leucyl-glycine
	leucyl-alanine
	tryptophan
	valylvaline
	prolyl-leucine
	asparaginyl-proline
	phenylalanylalanine
	prolylglutamic acid
	isoleucyl-isoleucine
	gamma-glutamylcysteine
	prolylphenylalanine
	phenylalanylvaline
	tyrosyl-L-proline
	leucyl-phenylalanine
	tyrosylleucine
	glutathione
	phenylalanylphenylalanine
	leucyl-tryptophan
	Ile-Pro-Ile
	viscumamide
Terpenoids	sabinene
	safranal
	citral
	geraniol
	geranylacetone
	loliolide
	nerol acetate
	caryophyllene
	cadinene
	cadinol
	vomifoliol
	genipin
	dehydrocostuslactone
	curcumol
	phytol
	acerogenin G
	terpineol
	hirsutanone
	roseoside
	lupane
	lupeol
	betulin
	betulinic acid
	β-amyrin acetate
	astragaloside IV
Phenolic acids	salicilyc acid
	cinamic acid
	gentisic acid
	p-coumaric acid
	gallic acid
	caffeic acid
	veratric acid
	ferulic acid
	syringic acid
	chlorogenic acid
	rosmarinic acid
Fatty acids	octanoic acid
	pentadecanoic acid
	palmitic acid
	linolenic acid
	linoleic acid
	stearic acid
	9-OxoOTrE
	arachidic acid
	12,13-DiHOME
	lignoceric acid
	cerotic acid
Organic acids	tropic acid
	aconic acid
	ascorbic acid
	citric acid
	quinic acid
Nucleosides	thymidine
	cytidine
	uridine
	adenosine
	guanosine
Alcohols and esters	2-phenylethanol
	1-octene-3-ol
	cinnamic acid methyl ester
	viscumitol
	sinapic acid
Amines	choline
	histamine
	acetylcholine
Coumarins	coumarin
	6-hydroxy-4-methylcoumarin
	7-methoxycoumarin-4-acetic acid
Alkaloids	sparteine
	lupanine
	retamine
Lignals	pinoresinol
	medioresinol
	syringaresinol
Sterols	β-sitosterol
	daucosterol
Aldehydes and ketones	vanillin
	ionone
Miscellaneous	nonanolide
	heptadecane
	carotene
	coniferin

**Table 3:** The characteristic group frequencies attributed to different metabolites identified in *Viscum album*.

Metabolites	Wavenumber (cm^–1^)	Ref.
Amino acids, peptides, thionins	3400; 3330–3130; 2530–2760; 2080–2140; 1724–1754 1687, 1675, 1663, 1654, 1644, 1632, 1621, 1611, 1610–1660, 1500–1600;	[5]
Flavonoids	1734, 1703, 1634, 1627, 1580, 1522, 1460, 1440, 1410, 1367, 1315, 1255, 630 and 575	[86,87,88]
Terpenoids	2938.7, 1740, 1651, 810	[87,89]
Phenolic acids	1800–1650, 1734, 1720, 1627, 1522, 1440, 1410, 1420–1300, 1367, 1315, 1255, 1170–1100	[88]
Fatty acids	3020–3010, 2924–2915, 2855–2847, 2800–2900, 1746, 1710, 1250, 720	[90,91]
Organic acids	1255, 1378, 1440, 1410, 1376	[88]
Nucleosides	968, 1120, 1175, 1330, 1420, 1480, 1725, 3270, 3600	[92]
Coumarins	600–900, 1200–1000, 1028, 1254, 1450, 1608, 1627–1715, 1740, 1760, 2207–2210, 2963, 3362, 3381, 3399, 3434,	[93,94]
Alkaloids	630–650, 925, 1200,1285, 1330, 1531, 1548, 1600–1650, 1653, 1658–1567, 1600, 1660, 1700, 1710, 3000, 3377, 3380, 3332, 3384.84	[95]
Sterols	740.5, 1062.5, 1192, 1383, 1465.6. 1737.5, 2937	[96]

## Data Availability

Not applicable.
